# Gallbladder perforation due to the obstructing periampullary duodenal diverticulum (Lemmel’s syndrome): a case report

**DOI:** 10.1093/jscr/rjaf129

**Published:** 2025-03-12

**Authors:** Shruti Sah, Nissant Subedi, Amit Shah, Pradeep Yadav, Narendra Pandit

**Affiliations:** Department of Gastrointestinal Surgery, Birat Medical College and Teaching Hospital (BMCTH), Tankisinwari, Morang, Biratnagar 56613, Nepal; Department of Gastrointestinal Surgery, Birat Medical College and Teaching Hospital (BMCTH), Tankisinwari, Morang, Biratnagar 56613, Nepal; Department of Gastrointestinal Surgery, Birat Medical College and Teaching Hospital (BMCTH), Tankisinwari, Morang, Biratnagar 56613, Nepal; Department of Gastrointestinal Surgery, Birat Medical College and Teaching Hospital (BMCTH), Tankisinwari, Morang, Biratnagar 56613, Nepal; Department of Gastrointestinal Surgery, Birat Medical College and Teaching Hospital (BMCTH), Tankisinwari, Morang, Biratnagar 56613, Nepal

**Keywords:** biliary stasis, duodenum, diverticulum, Lemmel syndrome, periampullary

## Abstract

Lemmel's syndrome is a rare condition characterized by obstructive jaundice due to the periampullary duodenal diverticulum in the absence of choledocholithiasis or tumors. Its infrequent occurrence and non-specific clinical presentation can make it difficult to distinguish from other conditions. We present a case of Lemmel's syndrome in a 63-year-old male who exhibited symptoms of abdominal pain in right hypochondrium, vomiting, and fever. Imaging studies (magnetic resonance cholangiopancreatogram, computed tomography scan, and endoscopy) revealed an oval-shaped duodenal outpouching, distended gallbladder, common bile duct dilatation, and surprising localised gallbladder perforation leading to the diagnosis. Surgical intervention, including cholecystectomy and choledochoduodenostomy, successfully relieved the symptoms and biliary obstruction.

## Introduction

Duodenal diverticula are uncommon outpouching of the duodenal wall, most commonly seen in the elderly. Approximately 70% are located in the periampullary region, with a smaller percentage found in the distal duodenum (D3 and D4) [1]. Duodenal diverticula are often asymptomatic, and are mostly diagnosed incidentally in up to 22% of the population, out of which <10% are symptomatic [2]. However, periampullary diverticula can lead to complications such as biliary obstruction, pancreatitis, bleeding, or even mimic cholecystitis [3, 4]. Lemmel syndrome, a rare condition caused by the periampullary diverticulum-induced biliary stasis, presents a diagnostic and therapeutic challenge [1, 5]. Here, we present a case of Lemmel syndrome with a rare complication of gallbladder perforation requiring surgical management.

## Case details

A 63-year-old male presented to the emergency department with a 3-days history of right hypochondrial pain, bilious vomiting, and fever. He denied any significant medical history. On examination, the patient was icteric, with stable vitals (PR: 90 bpm; RR: 20 breaths/min; BP: 110/70 mmHg). An ill-defined, tender lump (8 cm) was palpable in the right hypochondrium. The laboratory investigations revealed normal hemoglobin (11 gm%), leukocytosis (15 300/mm^3^; neutrophils: 89%) and deranged liver function tests (total bilirubin: 7.5 mg/dl, direct bilirubin: 3.5 mg/dl, alkaline phosphatase: 139 U/L, aspartate transaminase: 139 IU/L, aspartate aminotransferase: 277 IU/L). The renal function test and coagulation profile were normal. The ultrasonography showed a distended gallbladder (8.7 × 4 cm), dilated common bile duct (CBD) (18 mm), and mild intrahepatic biliary radicle dilatation, without any visible distal calculi or tumor.

**Figure 1:**
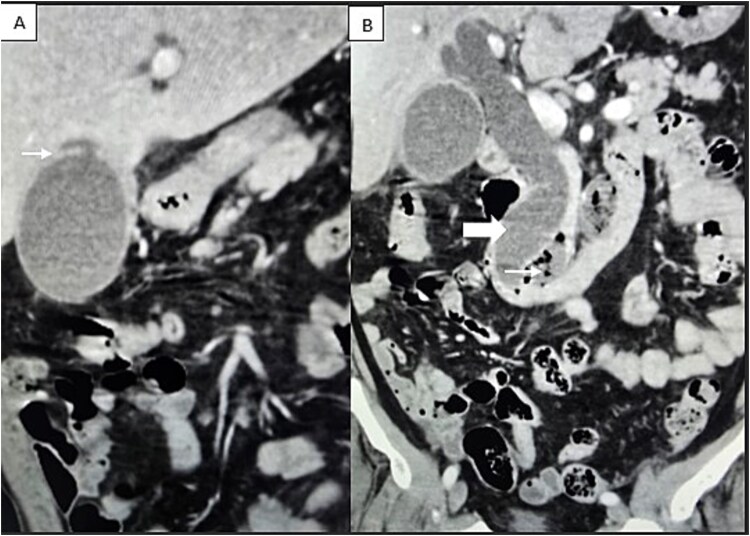
Contrast-enhanced CT scan demonstrating (A) localized perforation at the fundus of gallbladder (white arrow). (B) Extrahepatic biliary tract dilatation (big arrow); a periampullary duodenal diverticulum (small arrow).

**Figure 2:**
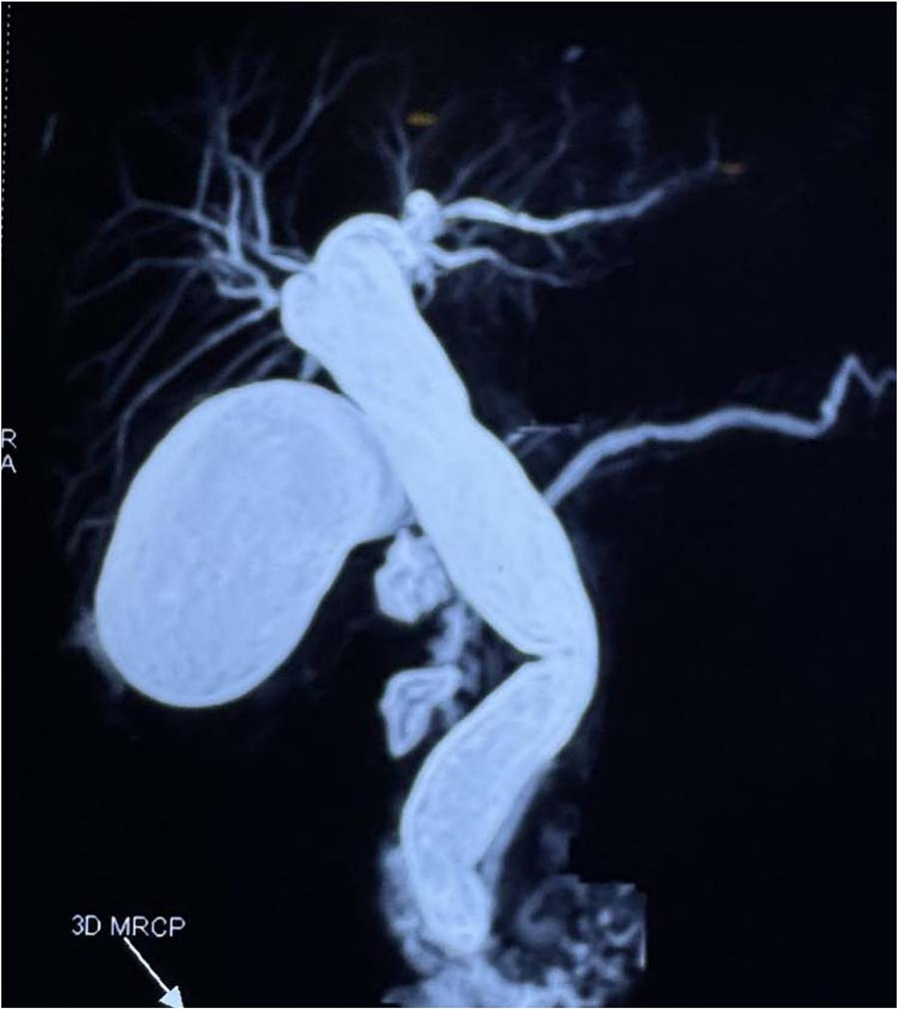
Magnetic resonance cholangiopancreatogram (MRCP) showing biliary stasis without any structural distal obstruction.

**Figure 3:**
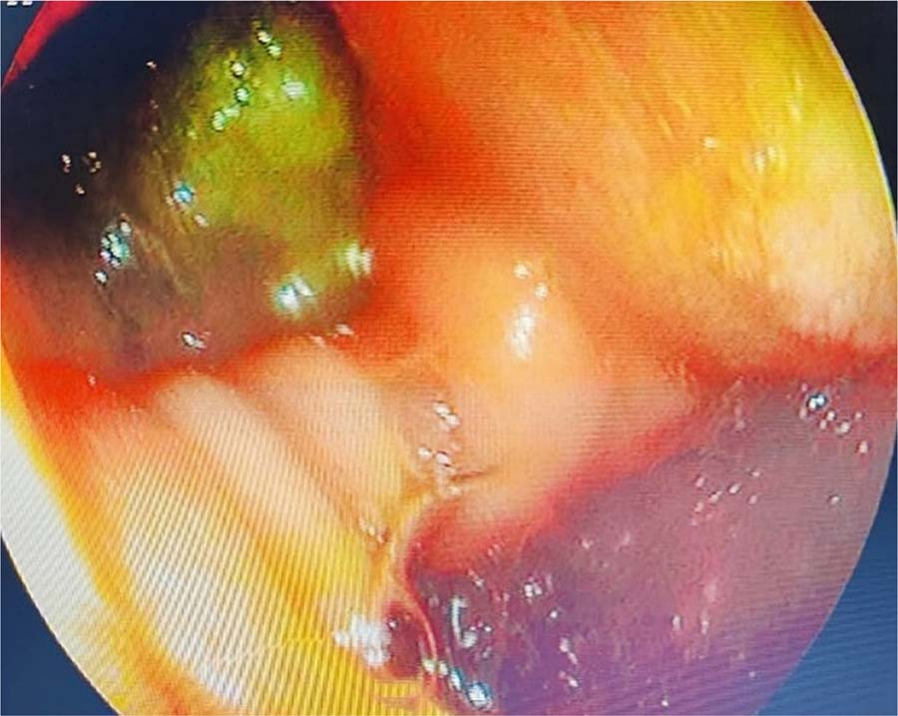
Upper gastrointestinal endoscopy showing duodenal diverticulum.

A computed tomography scan confirmed the presence of a duodenal diverticulum (3 cm) in the second part of the duodenum, localized perforation of the gallbladder fundus, and extrahepatic bile duct dilation (Fig. 1). Magnetic resonance cholangiopancreatogram further characterized the findings, revealing biliary stasis secondary to the periampullary diverticulum (Lemmel’s syndrome) without any structural distal obstruction (Fig. 2). Upper gastrointestinal endoscopy excluded periampullary malignancy and confirmed biliary stasis due to the periampullary diverticulum (Fig. 3).

In light of the patient’s clinical presentation (fever, leukocytosis, abdominal pain, and localized tenderness), and gallbladder perforation, surgical intervention was planned. At surgery, cholecystectomy, CBD exploration, and side-to-side choledochoduodenostomy were performed. The bilioenteric drainage relieved the distal obstruction due to the diverticuli. Given the patient’s advanced age and localized infection, the diverticulum was not resected to minimize surgical morbidity. Postoperatively, the patient had superficial surgical site infection. The jaundice improved, and so were the symptoms. The gallbladder histopathology was suggestive of xanthogranulomatous cholecystitis. He was discharged on Day 10. At 2-months follow-up, he is doing well.

## Discussion

Periampullary diverticulum are usually asymptomatic, and are an incidental findings at endoscopy. At most they cause epigastric burning type pain due to the retained food bolus in the diverticulum [2, 6]. Rarely, they become complicated with inflammation leading to perforation, bleeding and pancreatobiliary obstruction (Lemmel’s syndrome) [7]. Our patient had distal CBD obstruction due to a large diverticulum in the second part of duodenum, leading to biliary stasis and gallbladder perforation. Gallbladder perforation as a sequel of Lemmel’s syndrome has not been described in the literature. Due to the biliary stasis, gallbladder can be overdistended leading to mucosal ischemia, gangrene and perforation at the weakest point of gallbladder.

Treatment of such scenarios is cholecystectomy and distal biliary obstruction relief. Diverticulectomy in the second part of the duodenum is technically demanding and fraught with complications [8, 9]. The basic principle behind the treatment is ‘treat the patient, not the disease’ and often ‘less is more’. Hence, we opted for bilioenteric drainage by choledochoduodenostomy to relieve distal biliary obstruction without resecting diverticulum. If the patient is fit, and the diverticulum predominantly extramural, and on the non-periampullary, resection can be done if expertize is available [5, 8]. The alternative options includes endoscopic resection of symptomatic windsock diverticula, and can be performed in tertiary referral endoscopy centers [9, 10].

## Conclusion

Periampullary diverticulum leading to biliary obstruction is a rare entity. It can rarely present with obstructive jaundice and gallbladder perforation. In emergency, cholecystectomy with bilio-enteric bypass treated the patient with excellent outcome. This condition, while uncommon, emphasizes the need for careful evaluation in patients presenting with obstructive jaundice without evidence of choledocholithiasis or malignancy.
